# The silent epidemic of lymphogranuloma venereum inside the COVID-19 pandemic in Madrid, Spain, March 2020 to February 2021

**DOI:** 10.2807/1560-7917.ES.2021.26.18.2100422

**Published:** 2021-05-06

**Authors:** Laura Martínez-García, Mario Rodríguez-Domínguez, Clara Lejarraga, María Concepción Rodríguez-Jiménez, José María González-Alba, Teresa Puerta, Matilde Sánchez-Conde, José Manuel Hermida, Beatriz Romero-Hernández, Juan Carlos Galán

**Affiliations:** 1 Servicio de Microbiología. Hospital Universitario Ramón y Cajal and Instituto Ramón y Cajal de Investigación Sanitaria (IRYCIS), Madrid, Spain; 2 Centro de Investigación Biomédica en Red en Epidemiología y Salud Pública (CIBERESP), Madrid, Spain; 3 Centro Sanitario Sandoval, Hospital Clínico San Carlos, Instituto de Investigación Sanitaria San Carlos (IdISSC), Madrid, Spain; 4 Servicio de Enfermedades Infecciosas. Hospital Universitario Ramón y Cajal and Instituto Ramón y Cajal de Investigación Sanitaria (IRYCIS), Madrid, Spain

**Keywords:** lymphogranuloma venereum, COVID-19, sexually transmitted infections

## Abstract

Despite social distancing measures implemented in Madrid to prevent the propagation of SARS-CoV-2, a significant increase (57.1%; 28.5 to 38.5 cases/month) in cases of lymphogranuloma venereum was detected during the COVID-19 pandemic. This unusual scenario might have accelerated a shift in *Chlamydia trachomatis* (CT) epidemiology towards a higher proportion of L genotypes compared with non-L genotypes in CT-positive samples. Our data underscore the importance of surveillance of sexually transmitted infections during the pandemic, in particular among vulnerable populations.

In 2020, in response to the emergence and global spread of severe acute respiratory syndrome coronavirus 2 (SARS-CoV-2), different measures were undertaken in an attempt to control and stop the propagation of the infection. One of the measures widely used was strict social distancing with lockdowns [1]. Several studies have explored the impact of coronavirus disease (COVID-19)-related lockdowns on sexual practices [2,3]. The social changes reported could potentially decrease the risk of acquiring a sexually transmitted infection (STI). We aimed to address the impact of a national COVID-19-related lockdown in Spain during the pandemic waves on the diagnosis of new cases of lymphogranuloma venereum (LGV), an invasive STI caused by L genotypes of *Chlamydia trachomatis* in a large STI centre in Madrid.

## National health system responses to the pandemic waves in Madrid

A nationwide lockdown started in Spain on 14 March 2020 and, after 2 weeks, was extended for an additional 98 days to 21 June. Of note, a de-escalation strategy was started on 28 April, with differences between the autonomous regions of the country. In Madrid, one of the cities most affected by the COVID-19 pandemic, limited social contact (≤ 10 people) was permitted from 24 May. During the successive pandemic waves, different control measures were adopted, but restricted social gatherings were always allowed. The time periods for the three pandemic waves were defined as follows: first wave from 10 March to 10 May 2020 (the first COVID-19 cases were diagnosed at the end of February), second wave from 22 August to 22 November 2020 and third wave from 20 December 2020 to 20 February 2021.

## Evolution of the lymphogranuloma venereum epidemic

Our centre sees more than 25,000 patients annually, and is the only authorised centre for prescription of pre-exposure prophylaxis for HIV in Madrid, following 1,200 patients regularly. We analysed data from January 2020 to February 2021.

During the period of the pandemic from March 2020 to February 2021, a total of 284 LGV cases were diagnosed, of whom 51.1% (145/284) were HIV-infected patients and 98.9% (281/284) were men who have sex with men (MSM). An LGV case was defined in this study as a person with laboratory confirmation: among all *C. trachomatis*-positive samples, a real-time PCR based on *pmpH* gene deletion was used for detection of L genotypes associated to LGV cases [4]. We tested urine, urethral, pharyngeal and rectal swabs. The majority of LGV cases were detected in rectal samples.

**Figure 1:**
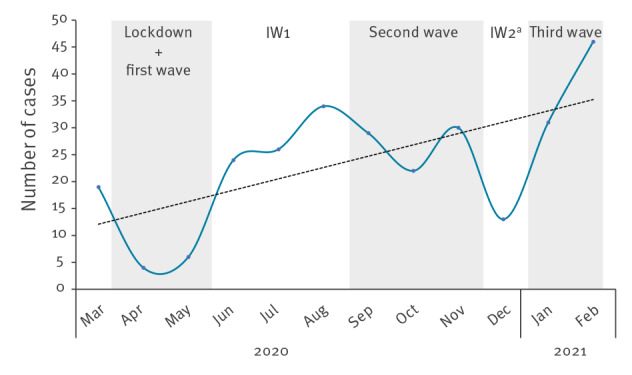
Evolution of lymphogranuloma venereum cases during the COVID-19 pandemic, Madrid, Spain, March 2020–February 2021 (n = 284)

COVID-19: coronavirus disease; IW1: interwave period 1; IW2: interwave period 2.

^a^ Analysis of IW2 was not performed because of a short timeframe.

**Figure 2:**
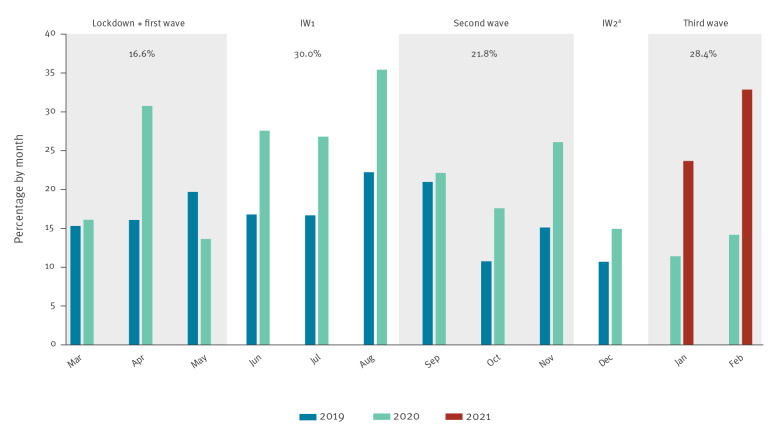
Monthly percentages of lymphogranuloma venereum cases with respect to *Chlamydia trachomatis-*positive samples by year, Madrid, Spain, March 2019–February 2021 (n = 536)

IW1: interwave period 1; IW2: interwave period 2.

^a^ Analysis of IW2 was not performed because of a short timeframe.

In the first pandemic wave, coinciding with the strict lockdown, the number of cases decreased dramatically compared with the previous 2 months (24.5 cases/month to 9.7 cases/month) (Figure 1). However, the proportion of LGV cases with respect to the positive samples for *Chlamydia trachomatis* (CT) during this period (16.6%, 29/175) was higher compared with the pre-lockdown period (13.1%; 49/375; 7 January–14 March) (Figure 2). In the period between the first and second wave (June–August 2020), the number of LGV cases increased rapidly, reaching an average of 28 cases per month, with a proportion of 30.0% LGV-positive of the CT-positive samples (84/280).

Blue line: number of lymphogranuloma venereum cases; dashed line: trend line.

The percentages represent the average of each pandemic wave period.

During the second wave, the number of LGV cases remained high, with an average of 27 cases per month, and the LGV-positive per CT-positive proportion was 21.8% (81/371), which was slightly lower than in the interwave period. The period between the second and third pandemic wave was very short, and we were unable to quantify the impact of LGV. During the third wave, a notable increase in the number of cases was observed (38.5 cases/month), reaching 46 new diagnoses in February 2021, which was the highest number observed during the pandemic. Moreover, the percentage of LGV cases with respect to the CT-positive samples also reached one of the highest values during this month (32.9%, 46/140). Comparing the first months of the study (coinciding with the pre-lockdown period) with the same months in 2021 (the last period of the study), the number of cases increased from 24.5 to 38.5 per month. In addition, we observed a significant increase in the percentage of LGV cases, evolving from 13.1% to 28.4% (chi-square test; p < 0.00001). Furthermore, it is also of note that the proportion of LGV to CT after the lockdown was always higher than in 2019 (Figure 2).

## Ethical statement

This study was approved by the Ethics Committee of Hospital Universitario Ramón y Cajal (Madrid) (Reference 012/17).

## Discussion

The decrease in the number of LGV cases during the strict lockdown is in agreement with other studies [5]. This outcome was probably related to the Spanish government’s decision to minimise all healthcare activities that were not dedicated to COVID-19 in primary care centres, such as STI clinics. As other authors have reported, this led to a reduction in the number of consultations [6] and in testing volume [7], since only patients with LGV symptoms were able to access the centres. In fact, a similar drop has been reported for a wide range of communicable diseases [5]. During this period, patients may have postponed visiting any medical centres, which may have caused more severe symptoms and additional complications in the absence of treatment [8]. This consequently provided more opportunities for transmission of CT infection, facilitating the spread of LGV. Moreover, a European study suggests that access to STI testing for vulnerable populations may have been reduced during the pandemic [7]. As expected, a reduction in the number of sexual partners [9] and sex frequency [10] has been reported; of note, an increase by 27.0% in the use of mobile sex apps during the strict lockdown was also detected [11]. These data could explain the significant increase in the number of LGV cases and the proportion with respect to the CT-positive samples following the first wave. Nonetheless, the resumption of social gatherings, including sexual activities, together with an increase in the asymptomatic screening in the re-opened STI centres [6] might also have led to the subsequent increase in LGV cases after the lockdown. However, the clinical activity in our centre increased progressively and, now in 2021, has reached the same levels as in the pre-pandemic period. During the second and third waves, the measures were less strict, as in other countries [12]. In Madrid, although social contact was still limited, gatherings up to 10 people were continuously allowed, which may explain why LGV incidence remained high. However, throughout the different periods of the pandemic, a progressive increase was observed, reaching concerning levels in the final days of the third pandemic wave. The proportion of LGV of total CT during first pandemic wave was higher compared with the pre-lockdown period. This is probably because patients infected by L genotypes are more likely to have symptoms than patients infected by non-L genotypes [13], and therefore are among the only patients to receive care during the strictest lockdown. Nonetheless, it is important to note that the proportion of LGV represented 32.9% of all CT infections during February 2021, highlighting that the LGV epidemic seemed to be challenging to control, continuing its spread and replacement of non-invasive CT genotypes among vulnerable populations.

## Conclusion

Our data revealed that the restrictions (including lockdown) have had no impact on reducing LGV transmission. It is important to make efforts to improve LGV diagnosis and surveillance, focusing on high-risk groups, such as MSM and other populations participating in dense sexual networks both during and after the COVID-19 pandemic.
